# Multiple virtual screening approaches for finding new Hepatitis c virus RNA-dependent RNA polymerase inhibitors: Structure-based screens and molecular dynamics for the pursue of new poly pharmacological inhibitors

**DOI:** 10.1186/1471-2105-13-S17-S5

**Published:** 2012-12-07

**Authors:** Mahmoud ElHefnawi, Mohammad ElGamacy, Mohamed Fares

**Affiliations:** 1 Informatics and Systems Department, Biomedical Informatics and chemoinformatics group, Division of Engineering Research and Centre of Excellence for Advanced Sciences, National Research Centre, Tahrir Street, 12311 Cairo, Egypt; 2 Science and Technology Research Centre, American University in Cairo, AUC Avenue, P.O. Box 74, 11835 New Cairo, Egypt

## Abstract

The RNA polymerase NS5B of Hepatitis C virus (HCV) is a well-characterised drug target with an active site and four allosteric binding sites. This work presents a workflow for virtual screening and its application to Drug Bank screening targeting the Hepatitis C Virus (HCV) RNA polymerase non-nucleoside binding sites. Potential polypharmacological drugs are sought with predicted active inhibition on viral replication, and with proven positive pharmaco-clinical profiles. The approach adopted was receptor-based. Docking screens, guided with contact pharmacophores and neural-network activity prediction models on all allosteric binding sites and MD simulations, constituted our analysis workflow for identification of potential hits. Steps included: 1) using a two-phase docking screen with Surflex and Glide Xp. 2) Ranking based on scores, and important H interactions. 3) a machine-learning target-trained artificial neural network PIC prediction model used for ranking. This provided a better correlation of IC50 values of the training sets for each site with different docking scores and sub-scores. 4) interaction pharmacophores-through retrospective analysis of protein-inhibitor complex X-ray structures for the interaction pharmacophore (common interaction modes) of inhibitors for the five non-nucleoside binding sites were constructed. These were used for filtering the hits according to the critical binding feature of formerly reported inhibitors. This filtration process resulted in identification of potential new inhibitors as well as formerly reported ones for the thumb II and Palm I sites (HCV-81) NS5B binding sites. Eventually molecular dynamics simulations were carried out, confirming the binding hypothesis and resulting in 4 hits.

## Introduction

It takes too long and costs too much to develop a new drug. Therefore, drug repositioning efforts are gathering more attention (i.e., to screen available drugs for new uses). Currently, fifty plus drugs have been repositioned http://www.drugrepurposing.info/. Off-label uses of drugs are widespread and legal in the USA. Also, multi-targeting compounds have been used in various diseases (e.g., receptor-thyrasine kinase inhibitors for various cancers such as GleeVec and Nexavir [1,2]).

This study presents a workflow for virtual screening and its application to Drug Bank screening targeting the Hepatitis C Virus (HCV) RNA polymerase non-nucleoside binding sites. Potential polypharmacological drugs are sought with predicted active inhibition on viral replication. Hepatitis C virus (HCV) infects over 3% of the world population and is one of the leading causes of chronic liver diseases [3]. About 80% of HCV-infected patients develop chronic hepatitis, 20% progress to cirrhosis and eventually develop Hepatocellular carcinoma [4]. Currently there is no vaccine available for HCV [5]. Current standard care of treatment for chronic hepatitis C is based on the combination of subcutaneous pegylated interferon-α and oral nucleoside drug ribavirin. However, serious side effects and poor response rates render the development of novel anti-HCV therapy an urgent need [3,6]. Several clinical trials are currently progressing for specifically targeted antiviral therapies (STAT-C) inhibitors that target specific protein pockets to inhibit HCV functions, while additional trials proceed on compounds which target host cell proteins that the virus utilizes for its survival/replication [7,8].

Currently, different targets for therapeutic intervention include structural as well as non-structural proteins and RNA structures in addition to post-transcriptional silencing. Non-structural targets include the NS3 protease covalent and non-covalent inhibitors, NS3-NS4A complex inhibitors, NS3 helicase inhibitors, NS4B inhibitors, NS5A inhibitors, nucleoside inhibitors and NS5B polymerase non-nucleoside inhibitors. These were recently discussed by Shimakami et al., [9] (and the included references). The RNA-dependent RNA polymerase NS5B in particular has been subject of intense research in the past decade because of its essential role in viral replication, its distinct features as compared to human enzymes, and ultimately due to its highly druggable nature [10].

**Figure 1:**
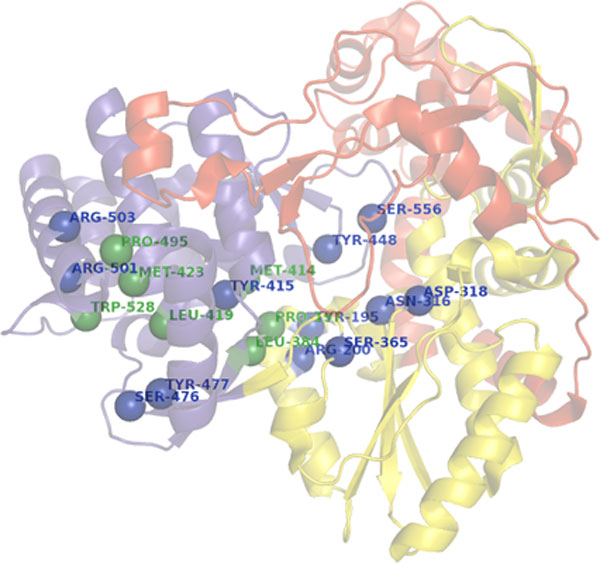
**A diagram showing the 3D structure of NS5B, showing key interaction residues (polar interactions; blue, hydrophobic; green)**. The three different domains are shown (thumb; blue, palm; yellow, fingers; red).

**Figure 2:**
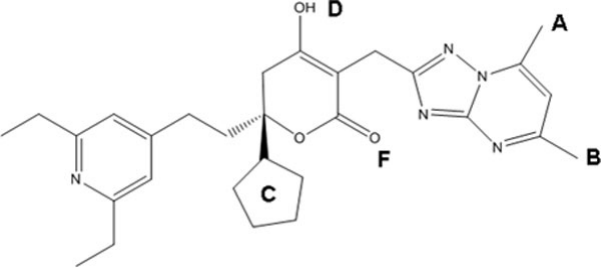
**A workflow describing the steps taken in both ligand-based and structure-based approaches to find novel inhibitors for HCV polymerase NS5B**. A) the ligand-based search consisted of pharmacophore generation and screening, followed by docking and selection.B) Structure-based NNI work flow consists of identifying the target binding sites and their interaction pharmacophore, a two-stage docking screen, combined with a neural-network ranking model for the hits, and finally, molecular dynamics simulations for the promising hits.

**Figure 3:**
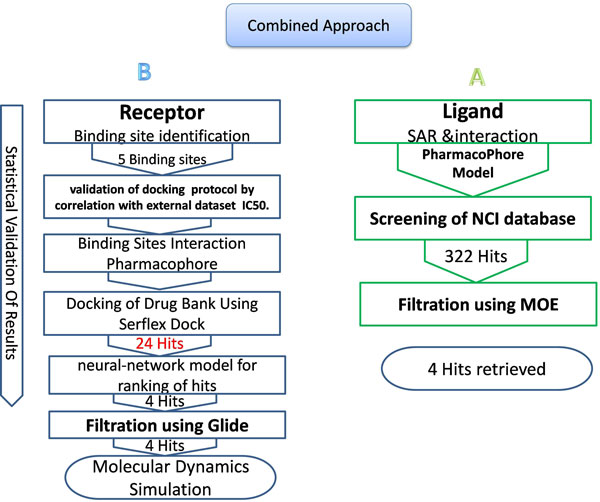
**Thumb II dihydro pyrol inhibitor (Filibuvir) pharmacophore used for screening several databases**. Its features are: A and B: two methyl groups and C: cyclopentyle group as well as D: one hydrogen acceptor E: The enol/ketone oxygen of the dihydropyrone and F: one other hydrogen donor and acceptor (The lactone carbonyl of the dihydropyrone).

**Figure 4:**
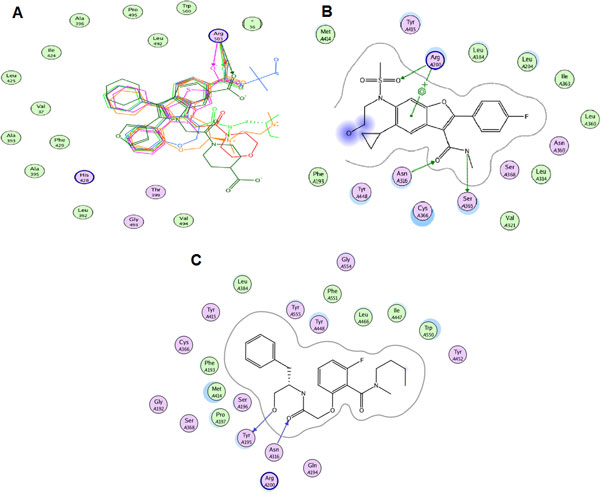
**Interaction pharmacophore of three NNI binding sites compiled from structural superpositionand alignment of relevant PDB coordinates for thumb I (A), palm II (B) and palm III (C), respectively**. The essential interactions shown are described in the results section. A) shows formation of hydrogen bond with ARG503 side chain and a hydrogen acceptor from the ligand B) HCV-796 inhibitor interacts with both side-chains of SER 365 -forms hydrogen bond with a hydrogen donor- and ARG200, in addition to arene-cation bond between the latter and the aromatic benzene ring of the benzofuran nucleus and H-bond donar with ASN 306 (not common for all pdb) C) The pocket seems to be narrow and only two polar interactions were computed with residues: ASN316 and TYR195, showing a tightly closed proximity contour with almost no solvent exposure by the ligand.

**Figure 5:**
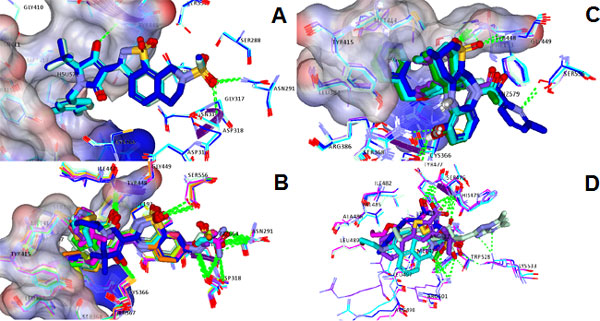
**Interaction pharmacophore for the Palm I subdomain**. Overlay of PDB coordinates of the three major chemical classes of NS5B palm I inhibitors A) class I (Benzothiazoles), B) class II (Benzo thiadiazines), C) class III (Benzodiazepines)). D) Overlaid complexes at the thumb II site (green dotted lines show polar interactions, different coordinates are colored differently, partial receptor surface is colored according to the interpolated charge-showing the whitish neutral regions)(polar hydrogens displayed in section C) all classes for Palm I shown in A, B, and C fill the deep hydrophobic pocket and shared TYR448 as a backbone HB acceptor. A) ASP318 also had polar interactions at the backbone. SER556 and ASN291 had hydrogen bond (HB) interactions through the terminal hydroxyl and amide groups with all members of class I.B) ASP318, GLY449 and ASN 291 were also involved in the same manner in addition to the terminal polar groups of SER 556 and CYS 366.C) shows that replacing the ketone on the hexene ring by a sulphone group expands the hydrogen bonding from TYR448 to TYR448 and GLY449, additionally, SER 367 HB seemed a common feature and to a lesser extent SER368. D) The main residues forming common polar interactions were SER476, TYR477 -as backbone HB acceptors and ARG 501 through the guanidinium group. A well defined π stacking was noticed between the histidine's imidazole ring and the filibuvir's (3FRZ ligand) triazolo pyrimidine group.

Although NS5B has the right-handed fingers, thumb and palm domains typical of polymerases, extensions of the fingers and thumb lead to a more fully-enclosed active site [11] (Figure 1). The inhibitors of HCV NS5B polymerase consist of two main classes: nucleoside inhibitors (NI) and non-nucleoside inhibitors (NNI) [12]. The NIs bind to the active site of the polymerase such as GS-7997, RGB7128, TMC649128, PSI-7977 and PSI-938. They currently offer the best candidates for cross-genotypic coverage and low resistant mutants. NNIs are a structurally and chemically heterogeneous class and do not induce premature termination of the RNA ssynthesis [13]. Moreover, NNIs are almost invariably allosteric inhibitors believed to block the enzyme, preventing a conformational transition needed for initiation of RNA synthesis [14]; the fact that corresponded with the results of Corbeil et al., [15] that assumed a solvated, and essentially flexible receptor [16]. These NNI classes bind to one of the four allosteric binding sites within the NS5B polymerase (Figure 1) [17] including: Site I (Thumb I) for JTK-109, benzimidazoles and Indoles [18], Site II (Thumb II) for dihydropyrols, phenylalanine analogs and thiophenes (PF-868554, VCH-759, VCH-916 and VCH-222), Site III (Palm I) for chemically heterogeneous leads such as ANA-598, A-848837 and ABT-333, Site IV (Palm II) for benzofurans as HCV-796 [19] and Site V (palm III) as phenylpropanyl benzamides [20]. For details, refer to the methods and results sections below and Figures 1,2, 3, 4, and 5 for a schematic of the NS5B polymerase and is important residues for each NNI site in addition to the minimum interaction pharmacophore for some major classes of NNI inhibitors.

**Figure 6:**
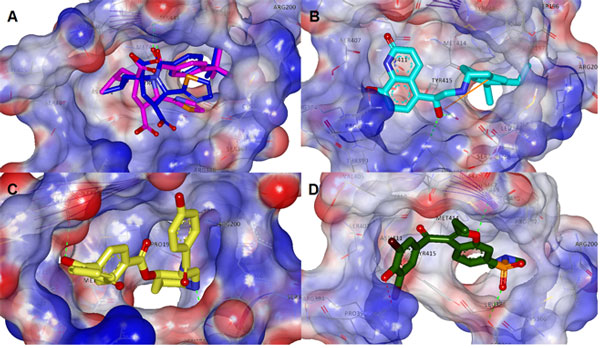
**A) Overlay of docked and PDB coordinates of 2JC1 ligand for the Palm I site (original ligand pose coloured blue, docked ligand pose coloured pink); B) docked pose of DB05039 it shows a very good placement of the diethyl-indanyl group into the deep pocket**. A salt bridge strengthens the binding with ARG394. Similarly, hydrogen bonding with ARG386 and TYR415, and Pi stacking with TYR415; C) docked pose of DB01940 forming a HB inside the deep hydrophobic pocket (as with one of benzodiazepines) in addition to a HB with GLN446 (just four atoms away along the backbone's TYR448 amidic nitrogen); D) docked pose of DB04142.(red dotted lines represent salt bridges, green dotted lines represent hydrogen bonds, orange solid lines represent π-interactions, backbone shown as curved purple lines, protein transparent surface with interpolated charges; bluish(+ve), reddish(-ve)). The furan ring show similar interaction with TYR448. These three hits preserved these binding modalities after molecular dynamics simulations.

Virtual Screening is the computational analogue of High Throughput Screening. It is defined as the *in silico *evaluation of properties, such as activity, or physiochemical properties like drug-likeness of different molecular scaffolds. Different applications of machine learning to virtual screening have been presented in the literature including both ligand-based similarity searching and structure-based docking. The main purpose of such applications is to prioritize databases of molecules as active against a particular protein target. *In silico *approaches such as virtual screening and structure-based design have emerged as a reliable, cost and time-saving technique complementary to in vitro screening for the discovery and optimisation of leads and hits. VS can be divided into ligand-based, structure-based and mixed approaches such as the approach implemented here (Figure 2). Activity-prediction/ranking models could be based on the set of ligands only which would be a purely ligand-based approach (such as the pharmacophore for thePF-868554ligand set pharmacophore built and shown in Figure 3). Or, it could be based on the 3d structure of the ligand-receptor complex (interaction pharmacophore) (such as those shown in Figures 4, 5, 6 for the different NNI sites). The same holds true for screening models which could be based on the ligand pharmacophore/3D quantitative structure-activity relationship (3D QSAR); or it could be based on the score of binding to the receptor (docking-based screen (as employed here).

Attempts to perform focused screening on specialised databases have been implemented before. These databases include some whose compounds have acceptable pharmacokinetic/pharmacodynamic properties (absorption, distribution, metabolism, excretion and toxicology) (ADMET) properties. Virtual screening of physiochemical properties as a first filter before activity-based screening has also been highly recommended in virtual screening protocols. It has been highlighted as a means of preserving time, and money. This has been triggered after the incompletion of a high percent of drug discovery projects with good activity due to problems with ADMET properties. Several studies have since indicated the importance of such prefilters and considerations of ADMET properties from the beginning. The SOCA approaches made use of focused libraries of well-studied compounds in terms of their pharmacokinetics for screening. Savarino et al., for example [21] using already available drugs as multivalent compounds for other diseases has been exploited for the HIV virus [22], for example the chlorophyll and the or auranofin gold nanoparticles. This has enlightened us to perform this study for HCV. Samewise, several *in silico *studies have been carried out previously from different perspectives to explore drug promiscuity. Keiser et. al., [23] has used molecular similarity measures to find new targets for old drugs with experimental validations. Also, microarray profiles of drugs have been successfully used for drug repositioning efforts to new diseases [24]. Here, a structure-based docking approach is used to find promiscuous drugs/compounds from the drug bank that could target the HCV polymerase allosteric sites. This short cut approach has yielded candidate hits that can immediately enter into clinical trials for dosage determination with minimal cost, pharmacokinetic, and toxicological profiling studies, which could offer a potential of improving treatment outcome with HCV chronic patients [25,26].

Both Ligand-based and receptor-based drug design approaches have been heavily implemented in finding new candidate inhibitors for HCV polymerase [11,14,27-32]. Yet to date, a comprehensive docking-based virtual screening of the Drug Bank for finding novel multivalent compounds has not been performed, although several studies reported the use of high throughput docking for lead identification and optimisation [14,16,33]. Furthermore, the combinations of docking tools that are based on higher accuracy scoring functions such as the XP (extra precision) and constrained docking approach in Glide were used to filter off potential false positives from the initial screening (Table 1). This was followed by molecular dynamics-based investigation of binding profiles of the resulting hits as detailed below.

**Table 1:** Docking scores and interactions for all NNI binding sites of hits structure against polymerase receptor with glide program using extra precision mode (hits with asterisk were selected for the molecular dynamics stage).

NNI Site	XP score	Pi interactions	Salt bridges	HB	Compound
Palm region	-8.5195	TYR415 (π-π)	ARG394	ARG386 TYR415	DB05039*
Palm region	-8.27362	-	-	GLN446 SER368	DB01940*
Palm region	-8.07386	-	LYS155 ASP220	CYS366 SER367 ARG158	DB00560*
Palm region	-7.11021	-	-	SER368 CYS366 GLN446	DB04118
Palm region	-7.0822	-	ARG394	SER368 TYR448	DB04142*
Palm region	-7.37393	-	-	ASP318 TYR448 ASN291	3D5M ligand*
Palm region	-6.93098	-	-	TYR448	2JC1 ligand*
Palm region	-6.5971	-	-	CYS366	DB01203
Palm region	-4.64441	TYR415 (π-π)	ASP318	GLN446 ASN316	DB01888
Thumb II	-7.80905	HIS475 (π-π)		ARG501 SER476	3FRZ ligand*
Thumb II	-6.9871	-	-	LEU497	DB00450
Thumb II	-6.28764	HIS475 (π-π)	-	SER476	DB04859
Thumb II	-6.01285	-	-	SER476	DB00481
Thumb II	-5.82026	-	LYS533 ARG501	TYR477	DB04205*
Thumb II	-4.39536	TRP528 (π-π) (2)	-	ARG501 LEU474 SER473	DB01087*
Thumb II	-3.17019	TRP528 (σ-π)	-	ARG501 LEU474	DB00816*

Validation of the docking and selection was performed in multiple steps. These included reproducing the original interactions of the reference enzyme-ligand complexes, comparing the root-mean square distance of the experimentally determined pose with the docked pose, and correlation of the enzymologic inhibition concentration (IC) 50/90 with the docking scores and sub scores. These validations for choosing the higher accuracy score for filtration were performed on datasets of known NNI binding sites inhibitors obtained from the Binding DB [34] in order to use the highest correlating score in the filtration of initial hits. Extending this idea further, here, we describe a neural-network artificial intelligence model that was constructed to provide better correlations of docking scores with experimental data through a target-trained model. The model is based on a multitude of scores and sub-scores from different scoring functions for each binding site. These are combined non-linearly via an artificial neural network classifier, that was used here for ranking the hits obtained from first-phase docking with Surflex (Table 2 and Additional files 1, 2, 3, 4). It could be later used after some statistical validations for screening massive compound databases. These multiple validation approaches were necessary in order to build confidence in the final predicted compounds to have novel inhibitory potential against the HCV polymerase. We are currently working on the experimental validation of these hits and extending this protocol.

**Table 2:** NS5B Palm inhibitors dataset from Binding DB training and correlation of different scores and of neural-network model (PIC).

Drugbank ID	Sybyl Total score	D_SCORE	PMF_score	G_score	cscore	Glide_xp_score	glide_constrained	MOE	IC50	"-Log IC50"	Predicted IC50
BindingDB_50139657	4.88	-111.015	-25.9954	-194.009	5	-6.18107	-7.46377	-11.2018	1000	-3	-3.405
BindingDB_50139665	3.38	-83.8017	-10.2113	-143.318	2	-5.72753	-7.42372	-12.3084	12000	-4.07918	-4.433
BindingDB_50139675	5.58	-106.188	-13.164	-158.077	5	-6.70348	-7.21041	-10.7057	2000	-3.30103	-2.717
BindingDB_50139676	5	-107.152	-11.3032	-158.959	1	-6.88072	-7.15086	-11.0469	1400	-3.14613	-3.09
BindingDB_50139677	4.27	-94.9065	3.964	-135.671	2	-6.40549	-7.13436	-11.3156	32000	-4.50515	-3.663
BindingDB_50139678	5.5	-114.532	-0.6873	-198.09	4	-6.76792	-6.89594	-10.9051	1500	-3.17609	-3.338
BindingDB_50139679	5.69	-104.815	-9.0235	-158.517	3	-6.1566	-6.88072	-11.313	50000	-4.69897	-5.371
BindingDB_50139680	4.95	-105.057	-12.5186	-145.938	5	-7.13436	-6.76792	-11.0915	34000	-4.53148	-4.862
BindingDB_50139681	4.64	-101.361	-2.2207	-151.314	2	-7.42372	-6.70348	-10.5949	2000	-3.30103	-2.717
BindingDB_50139682	5.37	-114.286	-22.6751	-146.53	5	-6.28327	-6.6221	-11.5046	1200	-3.07918	-2.157
BindingDB_50139683	5.08	-123.814	-25.821	-170.98	4	-7.46377	-6.41286	-11.1089	1200	-3.07918	-2.157
BindingDB_50139684	5.76	-109.545	-21.031	-175.899	2	-4.65519	-6.40549	-11.207	5000	-3.69897	-4.044
BindingDB_50139685	3.97	-121.737	-2.4307	-185.222	5	-7.21041	-6.28327	-11.1865	21000	-4.32222	-3.111
BindingDB_50139686	3.27	-113.558	-18.1067	-186.234	4	-6.41286	-6.18107	-11.4439	9000	-3.95424	-4.074
BindingDB_50139687	4.74	-96.1194	5.4162	-152.586	5	-6.89594	-6.1566	-11.2615	50000	-4.69897	-4.097
BindingDB_50139688	4.17	-107.649	-14.0725	-155.075	4	-7.15086	-5.72753	-10.883	3000	-3.47712	-3.65
BindingDB_50139689	5.4	-105.728	-12.6317	-155.943	5	-6.6221	-4.65519	-10.8427	50000	-4.69897	-3.964
**Pearson Correlation**	**0.184765**	**-0.42111**	**-0.50627**	**-0.35705**	**-0.09258**	**-0.08262**	**-0.35147**	**0.23712**	**-0.92744**	**1**	**0.68**

The model improved the correlation to 0.68.

Thus, through this work, novel inhibitors for the RdRp of HCV are sought. Combinations of ligand-based, receptor-based and incorporation of machine-learning classifiers were introduced along with molecular dynamics experiments to investigate the prospective inhibitors. Also, a meta-retrospective analysis to generate common contact pharmacophores that represent features required for efficient binding to NNI-sites for the HCV RdRp was performed by collecting all PDB files for each site, and finding common physical interaction moieties that are shared across all inhibitors of the same class that target that site.

For justification of our receptor-based approach, a ligand-based pharmacophore was built on a promising lead that is currently in clinical trials, Filibuvir (PF-00868554), targeting the thumb II site. This was used to screen different chemical databases with a few hits retrieved that need more activity and ADMET profile characterisation. These resulting hits were then short-listed using the docking approach. In the structure-based screening, a refined docking, ranking, and validation approach that employed machine-learning classification during the ranking process was performed for all sites of the RdRp on the drug bank database. The structure-based approach relied initially on virtual high-throughput docking of the drug bank on the four allosteric sites yielding tens of potential hits. This was followed by a second stage of more rigorous docking for the top candidates resulting from the former stage. Between them, ranking using the ANN model was applied. Also, validation using IC correlation for first-stage and rmsd for second-stage was done. Hit binding analysis selecting top poses and use of the interaction pharmacophores generated for each site followed. Further testing through molecular dynamics simulations culminated in potential hits acting on the palm I and thumb II were concluded that scored higher than threshold reference drugs, had low predicted IC values, and stable binding poses with molecular dynamics.

**Figure 7:**
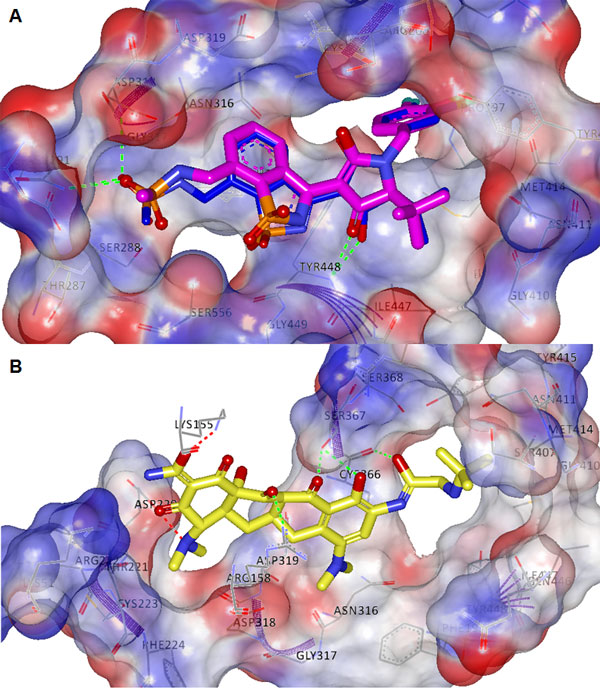
**A) Overlay of docked and PDB coordinates of 3D5M ligand(original ligand pose coloured blue, docked ligand pose coloured pink), B) docked pose of DB0056 showed H-bond donor with CYS366 and SER367 and h-bond acceptor with LYS 155 and ASP 319**.

**Figure 8:**
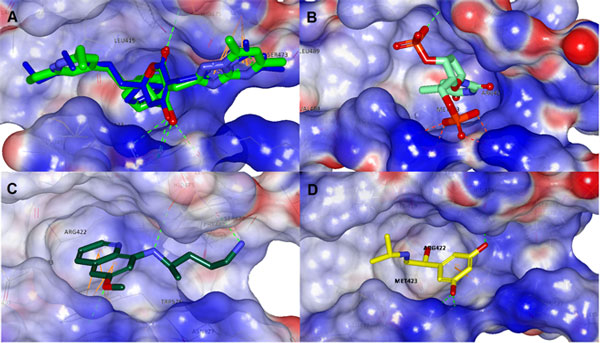
**A) Overlay of docked and PDB coordinates of 3FRZ ligand(original ligand pose coloured blue, docked ligand pose coloured green) for the thumb II site, B) docked pose of DB04205 it showed salt bridging with ARG501 & LYS503; C) docked pose of DB01087 it showed salt bridging with ARG501 & LYS503; D) docked pose of DB00816 it showed salt bridging with ARG 501 & LYS503**.

**Figure 9:**
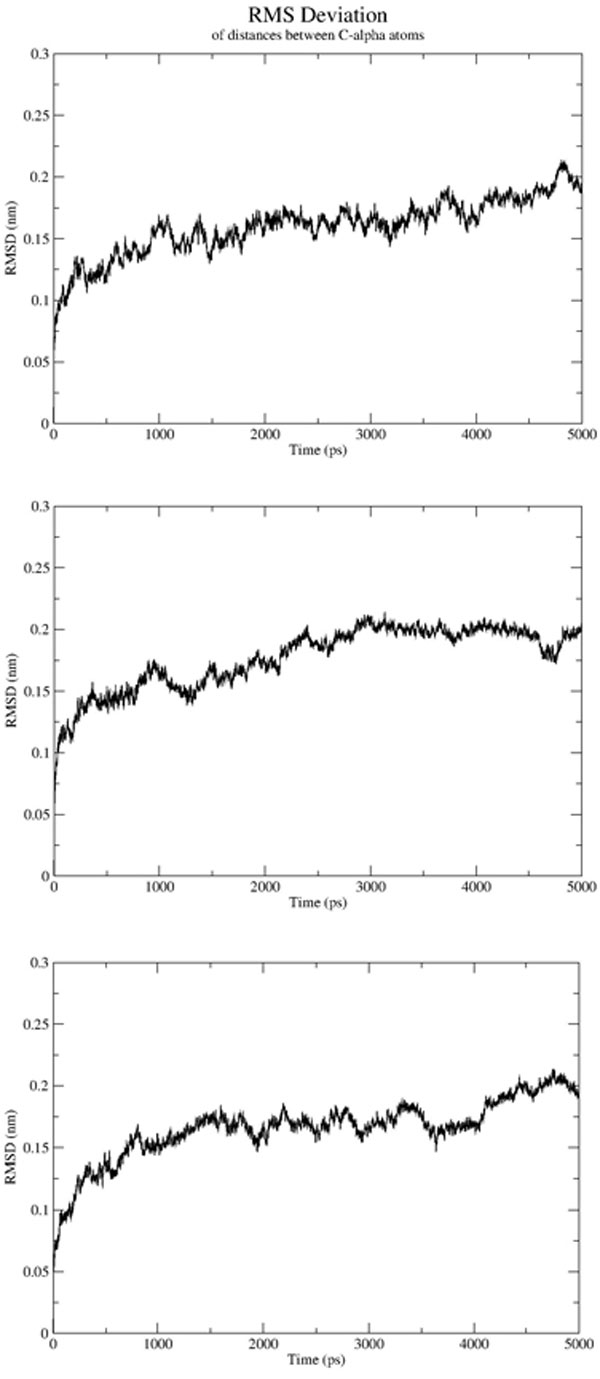
**RMSD across time along the production phase for the three protein ligand systems of the three PDB coordinates in the order: 2JC1 (palm), 3D5M (palm), 3FRZ (thumb II) (top to bottom)**.

**Figure 10:**
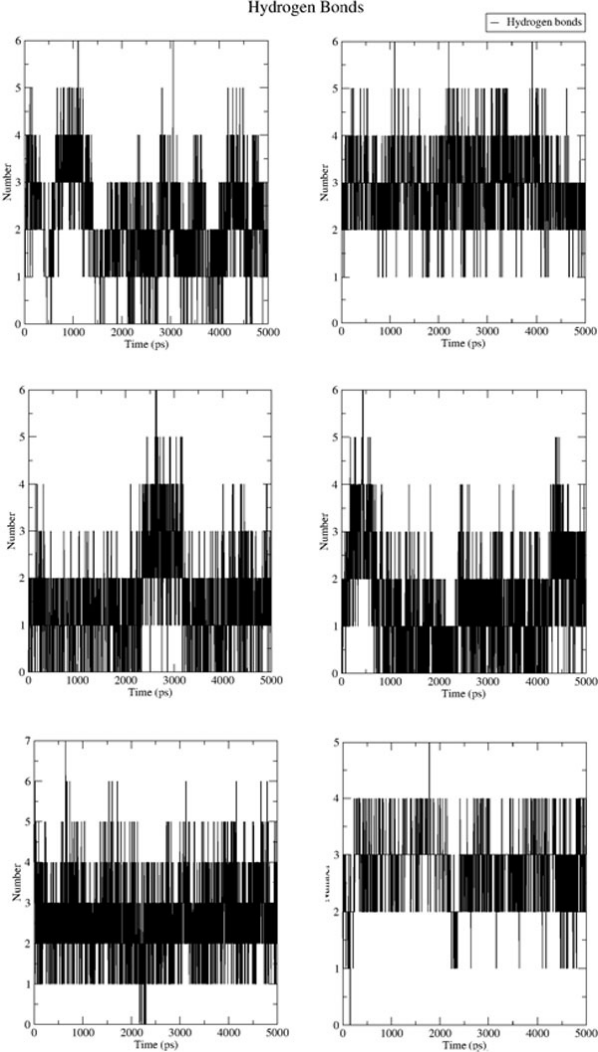
**Hydrogen bonds count formed between the ligand and receptor molecules across the production phase trajectory**. The plots represent the systems of NS5B structure complexed with the original PDB ligand coordinates and docked hits coordinates (at the palm region) in the order: 2JC1, 3D5M, DB05039, DB01940, DB00560, DB04142 (left to right, top to buttom).

**Figure 11:**
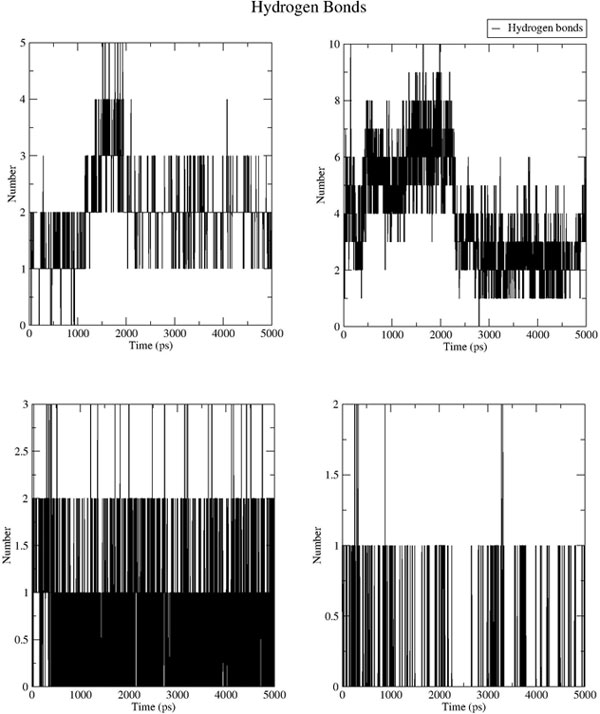
**Hydrogen bonds count formed between the ligand and receptor molecules across the production phase trajectory**. The plots represent the systems of NS5B structure complexed with the original PDB ligand coordinates and docked hits coordinates (at the thumb II region) in the order: 3FRZ, DB04205, DB01087, DB00816 (left to right, top to bottom).

The results and discussion sections will organise the work as follows: Figures 1,2, 3, 4, and 5. These show a schematic of the HCV polymerase (Figure 1), a schematic of the workflow (Figure 2), a pharmacophore for the thumb II site (Figure 3), and interaction consensus pharmacophores for all NNI sites (Figures 4 and 5). Other figures and tables illustrate the hits from different sites and their scores and modes of binding (Figures 6, 7, 8), Tables 1, 2 and Additional files 1, 2, 3 and 4. The final three figures show the molecular dynamics runs for the reference compounds and our potential hits (Figures 9, 10, 11). The ANN-models built using the external dataset and their applications are illustrated in Table 2 and Additional files 2, 3, 4.

## Methods

The flowchart in Figure 2 depicts the steps implemented in this study. Firstly, an interaction pharmacophore was generated for each NNI binding site to reveal essential interactions for each site. Secondly, a two-stage docking screen on the drug bank was implemented with increasing computational and time expense. A neural network model for predicting activity based on the docking scores was used for ranking the resulting hits. Finally, molecular dynamics simulations and comparison with the interaction pharmacophore was used for statistical validations of the resulting compounds. A ligand-based pharmacophore for the thumb II site (Figure 3) (which is a shallow site that could benefit more from a hybrid ligand and structure- based approaches) was used for searching diverse chemical banks and for comparison with the interaction pharmacophore.

### HCV polymerase allosteric binding-sites interaction pharmacophores generation

Finding the minimum requirements for efficient binding to all NNI-sites for the HCV RdRp was performed by collecting various PDB files for complexes at each site with different inhibitors, and finding common electrostatic/hydrophobic interactions that are shared across all inhibitors of the same class that target each site. Due to the large numbers of inhibitors used here, it was not possible to site all reference works of these inhibitors. However, we list the PDB codes and method of comparison below:

Thumb I: All available thumb I inhibitors (e.g. benzimedazoles [35]) crystal structures in PDB were obtained (PDB ID: 2BRK, 2BRL, 2DXS, 2WCX, 2XWY, 3MWW)

Palm I: Three main chemical classes were discerned for the Palm I site, nominally: beznothiazoles (PDB ID: 3D5M, 3H5S, 3H5U), benzothiadiazines (PDB ID: 3CWJ, 2FVC, 2GIQ, 3BR9, 3BSA, 3BSC, 3CDE, 3CO9, 3CVK, 3E51, 3G86, 3GYN, 3H2L, 3H59, 3H98, 3HHK) and benzodiazepines (PDB ID: 3CSO, 3GNV, 3GNW, 3GOL)

Palm II Site: The PDB files 3FQK and 3FQL complexes with the HCV-796 inhibitor in both coordinates.

Palm III site: obtained from the PDB file 3LKH.

Thumb II: Some of the crystal structures of polymerase and thumb II site inhibitors (PDB ID: 2HWH, 2HWI, 1YVZ, 3HVO and 3FRZ).

A protocol that utilizes Align123 to align protein sequences then superimposes the protein structures by the alignment (based on Cα carbons) was used to superimpose the PDB files (Average RMSD was approximately 0.6 Å) for common binding modes visualization of each site, with different PDB coordinates coloured differently. Analysis of the common hydrogen interactions, electrostatic interactions and hydrophobic common moieties was curated manually and revealed crucial information about the modes of binding of different classes of the HCV NS5B polymerase NNI inhibitors. These should be very useful for future HCV NS5B drug discovery efforts. The interaction pharmacophore for each site is illustrated below in the results section and shown in Figures 4 and 5. The structure-based docking protocol was used to check for the presence of those interactions in the highly scoring hits.

### Ligand preparation

Initially ligands were prepared using the 'Prepare Ligands' protocol in Discovery Studio 2.5 in order to standardize charges, enumerate ionization states and generate tautomers at physiological pH range (where chirality and general conformation were preserved). The ligands were typed using the CHARMM for partial charges set up all-atom CHARMM force field (version 35b1) (Momany-Rone partial charges method) [36]. The latter operation was followed by a minimization through 1000 Smart minimizer algorithm steps down to a RMS gradient less 0.05 kcal⁄mol⁄Å to 0.05 kcal/mol/Å in Generalized Born implicit solvent model [37] to filter out the energetically less stable ionic/tautomeric states, eventually selecting a single molecular structure per ligand. (Partial charges were replaced upon retyping at particular docking stages).

### Ligand-based screening

For finding Novel candidate inhibitors for HCV virus polymerase protein we took two different approaches: pharmacophore-based approach using a pharmacophore model built on the structure activity relationship of the guide leads, and a receptor-based approach by screening of the Drug bank on the different polymerase binding sites. The pharmacophore screening was performed on the thumb II site (Non-nucleoside inhibitor site), using the Structure Activity Relationship (SAR) of the experimentally active ligand set pf-00868554 [38], respectively. The built models were used in virtual screening against four different chemical databases: the NCI database [39], the Maybridge Hit-Finder [40], drug bank [41] and ZINC databases [42] using the MOE program.

Successful candidates were filtered using molecular docking; first, the receptor preparation was performed by addition of hydrogens and dehydration of the crystallization water molecules using the program default parameters. The docking was performed using "*triangle*" matcher as placement and "*londonDG*" for scoring and force field refinement. Identification of the binding site was performed using MOE's "Site finder" tool. ADMET properties were obtained for the highest scoring hits of the active site. Primary *in-silico *ADMET properties were predicted using the pharma algorithms ADME-TOX Boxes server (the server was free and latter commercialized). to determine Oral bioavailability, Absorption, Distribution, Solubility, And using Tox Boxes to determine Acute Toxicity (LD_50_, Mouse) and Acute Toxicity (LD_50_, Rat).

### Receptor-based screening

For finding Novel candidate inhibitors for HCV virus polymerase protein with acceptable ADMET profiles, our approach was screening of the Drug Bank database [41] for new candidates at the polymerase's different binding sites. For each site we have identified from the PDB five publicly available crystal structures of polymerase bound to different ligands representing the different binding sites (3MWW for an indole-based inhibitor [43], 2JC1 for Acyl pyrolidine inhibitors [44], 3D5M pyridazinone inhibitor [45] and 3FRZ [38] dihydropyran inhibitor) of the non-nucleoside inhibitors, mainly targeting the thumb I, thumb II and the palm region. Those four structures were used as reference leads to guide the screening protocol of the Drug Bank database using Surflex-Dock and also for validation of docking. The crystal structure files of polymerase NS5B co-crystallized with different ligands were downloaded from the PDB.

A two-phase docking was performed to filter the candidates, The first stage was performed using SYBYL X1.1 Surflex-Dock program(Tripose Inc) virtual screening mode [18] where the protein receptor was prepared by removing the unrelated substructure other than the ligand structure. Ligand structures were extracted and isolated in separate files. The side chains of the protein structure then were fixed using default settings, water atoms removed, hydrogen added, unknown atom types were assigned and bumps were relaxed. The Kollman-all atom charges were assigned to protein atoms. Finally, the whole structure underwent a staged minimization using the default parameters. The Surflex Scoring is based on the Hammerhead scoring function and a consensus score that is the linear combination of non-linear functions of protein-ligand atomic surface distances. The interactions include steric, polar, entropic, and solvation terms. In addition, a total score is also generated. At this stage scores were compared between the docked compounds and the original ligand's SYBYL total score. Only those with total score equal to or higher than the positive control's score for each site were accepted.

The potential candidates scoring higher than the control were subjected to the second phase of filtration using Glide [18]. Receptor pre-processing was performed by removing water molecules of crystallization among other pre-processing provided by "preparation and refinement" utility in Schrodinger package. The extra precision (XP) Glide method was used to dock the potential hits derived from SYBYL into their respective allosteric sites of HCV NS5B polymerase. The binding sites, for which the various energy grids were calculated and stored, are defined in terms of dimensions forming the enclosing box, which must contain all ligand atoms of an acceptable pose. They were defined according to the original inhibitor centroid and expanded to include any extra-residues that are reportedly significant for the respective site. Poses with a root-mean-square deviation (rmsd) of less than 0.5 Å and a maximum atomic displacement of less than 1.3 Å were eliminated as redundant to be able to increase diversity in the retained ligand poses. Poses retained for initial docking were doubled from the XP default (10,000) with extended sampling, also *in-situ *minimization rounds were quadrupled (400 steps) in order to increase the robustness and remove any unfavourable contacts in the final poses.

### Docking validation

Validation is the most important part for a successful drug design protocol. Several steps of validation were performed. The following methods were used:

1) The different scoring functions of used programs were correlated to the IC_50 _of experimentally determined inhibitor sets. So, we used structure sets from binding database [34] of known experimental activity. They were docked and scored using each scoring function. The correlation between the enzymologic IC_50 _of the structures and different scoring functions were calculated.

2) Another validation approach was performed by testing for the capability of the programs used to reproduce poses and interactions of sufficient similarity to the original crystal structure. This criterion was measured by calculating the root mean square deviation (rmsd) difference between the docked and the PDB coordinates of the co crystallised inhibitor for each site.

### Machine-learning target-trained model for efficient IC_50 _prediction

An Artificial Neural Network (ANN) model capable of predicting the IC50 for drugs targeting HCV polymerase thumb II binding site is proposed and implemented here. Chemical structure and IC_50 _of the PF-868554 and its related structures were obtained. Scoring of the compounds using various tools like SurFlex [18], Gold [19], Glide, and MOE followed. The features to build the classifier were based on the different docking scores and their components. The idea was to combine these scores non-linearly to attain a predicted activity. These experimental data were then split into training and validation datasets and as the training data was small we used cross-validation technique to evaluate our model, using WEKA version 3.7.1 (Waikato Environment for Knowledge Analysis, University of Waikato, and NZ) [46]. This model was then applied to rank and filter the virtual hits for this site based on (PIC50 of our candidate drugs and evaluate them. Due to the wide range variability of the IC50 between the drugs, we applied - log IC50 (PIC50) instead of IC50. We did a comparative study between various tools outputs including GOLD, GLIDE, MOE and SYBYL; to select the proper parameters to build our model, based on the correlation coefficient between each set of parameters and the experimental PIC50 (Table 2 Additional files 1, 2, 3, 4).

### Molecular dynamics

The GROMACS 4.5.4 package [13] was used for all of the simulation and analysis of the molecular dynamics experiments. Initially protein structures were cleaned from non-protein non-ligand atoms, then the topology files were generated for the protein and ligand separately using the GROMOS96 43A1 force field and the PRODRG tool [40] respectively. The system was created by manually including the ligand topology into the system's, followed by configuring a dodecahedron box of a margin of 1 nm in all directions, which was filled with water using the SPC explicit solvation model. The net charge of the system was then neutralized by adding chloride ions, thus rounding up to around 60,000 atoms per system.

Minimization was carried out through 100,000 steps with a convergence criterion of maximum gradient of 500 kJ/mol/nm by the steepest descent algorithm.

Force constant of 1000 kJ/mol/nm, two temperature coupling groups were created (protein and ligand, water and ions), and the modified Brendsen thermostat was hence used for both of the equilibration phases. Each of the NVT (where the system temperature was raised up to 300K) and NPT equilibration phases were 150 ps long (pressure coupling was isotropic, using the Parrinello-Rahman method).

For all of the runs (including the equilibration phases) the leap-frog integrator was used for force calculations with the Particle Mesh Ewald method used for electrostatics calculations, and time step of 2 fs. Finally, the production phase was conducted -where the system was fully unrestrained- for 5 ns for each complex, the trajectory of which was target of all of the analytical procedure reported herein. All these steps were performed using a multitude of tools available in the GROMACS package.

The production phase was computed through the MPI-aware version of MD run executable at a Sun Microsystems cluster, deploying 40 (8 threads each) nodes, accounting a net of 1.4 petaFLOP per run, kindly provided through the library of Alexandria super computer system.

## Results and discussion

Several drugs have been recently repositioned for other diseases in the market, with up to two thirds of the costs being cut during the drug discovery course since only phase II clinical trials were the starting point. The promiscuity of compound binding, and the multi targeting strategy are being explored for different purposes and the old paradigm of one key one lock is being changed. This could also be due to the limited space of folds and binding pockets in proteins that are chosen by nature, and the similarity of binding/different binding preferences that one compound can make.

### Pharmacophore-based virtual screening

In the first phase of our work, we consider only a ligand-based approach to find new candidates for HCV targeting the polymerase protein. The Structure Activity Relationship (**SAR**) obtained from "pf-008654" was used for building of the pharmacophore model for the thumbII site. The pharmacophore of the PF-868554 features are (Figure 3): the enol/ketone oxygen of the dihydropyrone which appear to form a direct hydrogen bond to the backbone amide NH of Ser-476 and a water-mediated hydrogen bond to the amide, also NH of Tyr-477 (the donor-donor motif). The lactone carbonyl of the dihydropyrone is involved in a water mediated hydrogen bond to Arg-501. The phenol functional group forms another hydrogen bond with Leu-497 through a water molecule, while the phenyl ring occupies an otherwise hydrophobic pocket. [Figure 3 shows the features of the pharmacophore on the PF008654 ligand].

The screening of the databases Drug bank, Maybridge and zinc databases yielded no significant structures while the NCI database yielded two significant hits. The hits were subjected to further analysis through docking with Surflex and Glide achieving significant docking scores. The thumb II best hits NSC 115863 and NSC 295688) which by docking scored (-18.803 and -16.119 respectively but with no hydrogen bonds for the NSC 115863 and one water bridge hydrogen bond for the NSC 295688). This motivated us to implement a more rigorous virtual screening method using structure-based design after understanding the essential binding requirements as shown below.

### Polymerase NNI binding-sites essential interactions pharmacophores

Inspection of the interactions and binding modes of different classes of molecules that have exhibited strong inhibitory activities to different HCV polymerase binding sites was carried out, which provides a better insight into the essential residue interactions for each NNI site (Figure 1 shows a 3D structure diagram of the NS5B domains and their key residues).

*Thumb I: *Ligand interactions were calculated and it was noticed that the residues that form the pocket are MET36, VAL37, ALA396, LEU392, ALA393, ALA395, THR399, ILE424, LEU425, HIS428, PHE429, LEU492, GLY493, VAL494, PRO495, TRP500, ARG503. Figure 4-A shows how important is formation of hydrogen bond with ARG503 side chain and a hydrogen acceptor from the ligand as all structure share the same interaction. In addition, resistance towards benzimidazole thumb I inhibitors was reported upon mutations at PRO495 position [47].

*Palm II: *The phamacophoric features were observed from the interaction of HCV-796 inhibitor with both receptor's side-chains of SER365 -forms hydrogen bond with a hydrogen donor- and ARG200, in addition to arene-cation bond between the latter and the aromatic benzene ring of the benzofuran nucleus (Figure 4-B). In 3FQK the ligand forms a hydrogen bond (HB) with ASN316 which is not present in the structure 3FQL due to replacement of ASN with CYS. This substitution seemed to significantly affect the binding, suggesting that HB an essential interaction (bearing in mind that position 316 already affords natural sequence polymorphism). The S365T mutation seemed to be equally disruptive to the K_d _(fold-shift > 200) as well. The pocket is formed of the following residues LEU204, LEU314, VAL321, LEU360, ILE363, SER365, ASN369 and the overlapped part with palm I site with residues MET193, PRO197, ARG200, ASN316, CYS366, SER368, LEU384, MET414, TYR415 and TYR448.

*Palm I: *From analysis of binding of the ligands of the aforementioned crystal structures, the ligands were categorized according to the three largest chemical classes of inhibitors co-crystallized with protein; class I (benzothiazoles), class II (benzothiadiazines), and class III (Benzodiazepines) which each bind to the palm I site in different modes. The site is at the junction of the thumb and palm domains and in proximity of the active site (similarly palm II and palm III), the pocket is formed of the following residues GLN184, MET193, PRO197, ARG200, THR287, SER288, ASN291, ASN316, GLY317, ASP318, CYS366, SER368, LEU384, GLY410, ASN411, MET414, TYR415, GLN446, ILE447, TYR448, GLY449 and SER556, these residues show partial overlap with palm II site in the following residues MET193, PRO197, ARG200, ASN316, CYS366, SER368, LEU384, MET414, TYR415 and TYR448.

From Figure 5 it seems obvious that all of the inhibitor classes fill the deep hydrophobic pocket, as also described as an important binding feature by Vandyck et al., [14]. It is also thought to afford the most substantial Van der Waal interaction energy contribution and confers an important selectivity character. Point mutations at MET414 [48,49] (which forms a major portion of the deep hydrophobic pocket) resulted in resistance against benzothiadiazines. On the other hand, there are diverse polar interactions among the classes in addition to few common ones. In particular, the amino acid TYR448 as a backbone HB acceptor has been proven to play a critical role regardless of the chemical class. This fact was established through molecular dynamics simulations showing the free energy decomposition for different residues performed by Li et al., [50] and by point mutation studies at 448 [49] which again resulted in benzothiadiazine-resistant mutants. ASP318 also had polar interactions at the backbone. SER556 and ASN291 had hydrogen bond (HB) interactions through the terminal hydroxyl and amide groups with all members of class I shown in Figure 5. For class II ASP318, GLY449 and ASN291 were also involved in the same manner in addition to the terminal polar groups of SER556 and CYS366. Lastly, for class III it seemed that replacing the ketone on the hexene ring by a sulphone group expands the hydrogen bonding from TYR448 to TYR448 and GLY449, additionally, SER367 HB seemed a common feature and to a lesser extent SER368.

*Palm III: *To date only a single PDB coordinate set was reported and deposited, showing the inhibitory potential of phenylpropyl-benzamides at the recently observed palm III. The key interaction residues were found to be TYR195, PRO197, ARG200, LEW384, MET414, TYR415, ASN316, ILE447, TYR452, LEU446, TRP550, and PHE551. The pocket seems to be narrow and only two polar interactions were computed (Figure 4-C) with residues: ASN316 and TYR195, the figure shows a tightly closed proximity contour with almost no solvent exposure by the ligand.

*Thumb II: *The thumb II site is clearly distinct from the thumb I site and each site has its separate residues, thumb II site is formed from the following residues (LEU419, ARG422, MET423, HIS475, SER476, TYR477, ILE482, VAL485, LEU497, LEU489, ARG501, TRP528, and LYS533), it lies at the base of the thumb domain around 35 Å from the active site (Figure 5-D). The main residues forming common polar interactions were SER476, TYR477 -as backbone HB acceptors- and ARG501 through the guanidinium group. A well defined π stacking was noticed between the histidine's imidazole ring and the filibuvir's (3FRZ ligand) triazolopyrimidine group. Other inhibitor-specific hydrogen bonding was noted by the residues LYS533, TRP528, ARG422 and MET423 (it is noteworthy that 419 and 423 mutations exhibited viral selection against thiophene-based carboxylic acid derivatives [51]). The inhibitor on which the ligand-based pharmacophore model was based (Figure 3)afforded direct polar bonding with SER476, ARG501 and Van der Waal interaction through the cyclopentyl moiety (with the shallow pocket formed of TRP528, MET423, LEU419 and TYR477) also π-π interaction with HIS475.

Based on this retrospective analysis, the analysis performed seemed to be consistent with results shown above from single point mutagenesis studies in confirming some predicted key interaction residues. Particularly important are ASN316, SER365, TYR448 and MET414 are at the palm region, LEU419 and MET423 at the thumb II site.

### Receptor-based screening

The screening of the drug bank on the three sites specified using Surflex-Dock yielded several potential binders; those compounds were subjected to our two-phase docking using SYBYL then Glide, as described earlier. In SYBYL we selected the ones exceeding the control threshold score (the experimentally proven ligands). Several candidates were found targeting the three sites on HCV polymerase, twelve compounds targeting NNI site I, ten compounds targeting NNI site II, five compounds targeting NNI site III, while for thumb I no significant hits were achieved from score prospective. On the second docking phase we used Glide extra-precision (XP) docking to further filter our candidates.

### First stage filtration with SurFlex

Different ligands of proven activity/affinity against the polymerase which came co-crystallized with polymerase receptor and bound to its different binding sites were separated and re-docked into polymerase protein. The mean of the docking scores of these ligands was used as a threshold for first phase filtration of the screening results of the drug bank on the different sites of the polymerase receptor. This process resulted in 84 high scoring structure ligands that exceeded the threshold of 8 of Surflex-Dock total score. They were arranged as 19 structure for thumb II site, 12 structure for palm I site, 52 structure for palm II site (Additional file 1) while no structures succeeded to cross the filtration threshold in thumb I, interestingly we noticed that 9 structures of high scoring drugs showed potential activity against more than one binding site which are (DB01166, DB01036, DB04859, DB00918, DB04471, DB01087, DB06202 and DB00481) two of these drug, DB01036 and DB04859 achieved high scores for three binding site which are (palm I, palm II and thumb II) with scores of (8.62, 8.03, 8.03 for DB01036) and (8.58, 8.02, 8.24 for DB04859) in the three binding sites respectively, also DB01166 appears to bind at the palm I site with a score of 12.16 while DB04471 appears to bind to palm II and thumb II with the scores 9.65 and 9.36.

Upon screening the drug bank, DB01166 achieved highest score; 12.16, then the compounds DB00777, DB04471, DB05039, DB02166 and DB04205 with scores 9.98, 9.36, 9.23, 9.19 and 8.83 respectively (Table 2).

On the other hand the further filtration process based on consensus of scores of many docking programs which were incorporated in one machine learning model (described in the methods section) has helped in the selection and correlation with enzymologic IC. According to the model results, the correlation was improved slightly using the model for the thumb II site (from 0.86 to 0.87) and significantly for the Palm I site (from 0.5 to 0.67 (Table 2 and Additional files 2,3, and 4). The model was then used for ranking the filtered hits based on the predicted IC values. The domain of applicability of the neural-network model here was used only for ranking, as the idea of a machine-learning model based on docking scores as features was not implemented before. We will seek to expand this model in terms of a docking-based workflow and machine-learning filtration in the future to be applied to diverse chemical databases. Many of these drugs have proven good affinity against RDRP. The DB01940 which achieved the highest score for palm I site driven from the model the potential binding mode proposed by surflex shows that the azocane ring of the structure fixes the structure to the pocket B while formation of 4 hydrogen bonds two of them are the same formed by the original ligand which are ASN411, Gly449 and two other hydrogen bonds with each of Tyr415 and Tyr448, while on the molecular fields level fair similarity of the hydrophobic molecular field to the original ligand was noticed. We were encouraged by the identification of some known HCV polymerase inhibitors among the retrieved hits (e.g.: HCV-086 in Additional file 1), which gave us more confidence in the work flow and methodology that we have proposed. Now, these eighty six filtered compounds were advanced to a second round of filtration as detailed below.

### Second stage filtration with XP Glide docking

While no potential final list candidates were found for the thumb I site; several candidates that fulfil the inhibitory pharmacophoric requirements for the thumb II site, and the palm region were found. The next phase of docking was performed using the Schrodinger Glide module version 5.5, in particular, implementing the CPU-expensive Glide XP docking function which has resulted in satisfactorily low RMSD differences upon re-docking the co-crystallized inhibitors (please refer to the validation title in the introduction point 2 for details). Consequently from the resultant hits only 7 compounds were selected according to their affinity and mode of binding for the final molecular dynamics simulation.

For the palm region, docking of the co-crystallized ligand resulted in a pose with a RMSD of 1.2 Å and 1.09 Å calculated from superposition of heavy atoms of the docked ligands over the crystal coordinates for PDB coordinates 2JC1 and 3D5M respectively. Through retrospective inspection, the top four ranking hits were selected for further analysis as they bare a relatively rich electrostatic interaction profile as well as sufficiently high Van der Waal interaction XP terms with the receptor. The rest of the hits showed very weakly interacting poses and Glide XP scores lower than that of the co-crystallized -reportedly potent- inhibitor [44,45] (except for DB04118 and DB01888).

The original ligand of 2JC1 (Figure 6-A), as in the crystal structure, had a docked pose exhibiting a single hydrogen bond with the TYR448 residue, which suggests the sufficiency of that bonding - in addition to filling the deep hydrophobic pocket with the *p*-tert-butyl phenyl group - as a criterion for inhibition at this site. Worth noting is the improved field positioning for the carboxyl group of the docked pose right amid the two guanidinium groups of ARG394 and ARG386, that might have happened because we haven't minimized the PDB structures before superimposition. In spite of the relatively low RMSD value for superposition of the docked and original poses of 3D5M ligand (Figure 7-A), the docking result gives a better preposition of a possible proton exchange between the sulphonamide of the ligand and the ASP318 carboxyl, and yet again hydrophobic moiety (the di halo-substituted phenyl in this case) fell very well into the deep pocket (Figure 7-A).

Though realizing the highest docking score, hit DB05039 did not interact with any of the common polar interactions discussed, yet it shows a very good placement of the diethyl-indanyl group into the deep pocket (Table 1). A salt bridge strengthens the binding with ARG394. Similarly, hydrogen bonding with ARG386 and TYR415, and Pi stacking with TYR415. DB01940 followed in terms of score, interestingly enough with an azepane ring forming a HB inside the deep hydrophobic pocket (as with one of benzodiazepines) in addition to a HB with GLN446 (just four atoms away along the backbone's TYR448 amidic nitrogen) (Figure 6-C). Hence, both of the formerly mentioned ligands were found to be of interest, and may potentially define new pharmacophoric features.

The hit DB00560 ranked third (Table 1) with a glide score of -8.073859 and achieved the highest electrostatic energy contribution of all with three hydrogen bonds and two salt bridges with the receptor, superseding the original ligands in terms of coulombic interaction term as well as total score, although it seems to possess a different positioning than the other hits, the structure was protonated from predocking processing at the secondary amine, which was the reason why the tert-butyl placement failed inside the hydrophobic pocket (Figure 7-B). Albeit the compound already satisfies two of the common interactions discussed, namely with CYS366 and SER367.

In comparison between the pose of hit DB04142 and that of the original ligand (Figure 6-D), we find a similar placement, similar TYR448 interaction, a significantly enriched electrostatic field complimentarity with the site features, even less unfavourable ligand solvent exposure, and obviously less expected conformational entropy. The docking score lied well between those of the two original ligands.

While for Thumb II the main trend of ligand-receptor interaction observed from the docked set was mainly comprised of the Van der Waal terms -the contributed to most of the total binding energy- with many unfavourable solvent-surface exposure, yet in the contrary to the palm region the hydrophobic of thumb II is quite shallow, the fact that down weighs such terms. Most of the set exhibited minimal electrostatic interaction (in most cases a single hydrogen bond was observed), nevertheless, π interactions were common (especially with TRP528, due its annular residue proximity).

An RMSD of 0.78 Å was obtained upon re-docking the original ligand of 3FRZ. The overlaid poses are shown in Figure 8-A where the docked pose seemed to have shifted slightly to form HB acceptor and donor -to TRP528 and ARG501 respectively- out of the hydroxyl group instead of a double acceptor -from ARG501.

Only 3 ligands (DB04205, DB01087, DB00816) were observed to possess better binding modalities (Figure 8), including salt bridging with ARG501 & LYS503, along with hydrogen bonding with the protein's backbone at different residues albeit minimal. Also π-π, σ-π interactions were observed (Table 1), while they recorded the highest coloumbic energy terms. The three compounds were candidates for further investigation though molecular dynamics simulations for a sufficiently long production phase. In spite of the prospected good binding properties of DB04205, it seems to be highly hydrophilic and thus not expected to be effective in vivo as is. So a pro-drug strategy is required during the biological assays. Also the stability of DB00816 inside the binding site was questionable due to improper positioning of group and the apparently promiscuous nature of the hit as a non-selective ligand due to the relatively small size.

From these two stages of docking screens and ranking, some interesting compounds have emerged that will be taken into molecular dynamics simulations as the next and final step in this protocol. These compounds belong to diverse structural classes, highlighting one advantage of the structure-based approach. Some of them also satisfy some of the interaction pharmacophoric features that were identified, while others show new binding modalities.

### Molecular dynamics simulations

For a stronger hypothesis and a better insight into the validity of the docking results achieved, we have conducted coarse-grained molecular dynamics simulations for the formerly mentioned selected group of ligand-protein complexes that were the result of the two-phase docking and selection phases using the GROMOS force field. It was performed in the form of 20 ns of unrestrained production phase dynamics, following a double equilibration period 300 ps long where constant temperature (298.15 K) and pressure (1 atm) were imposed. The C_α _RMSD was calculated and plotted for all of the systems starting from the end of the equilibration phase. Since all of the systems have produced similar plots in terms of time to RMSD stability tendency beyond the 2500 ps interval, Figure 9 shows C_α _RMSD for only the three original systems derived from the PDB coordinates taken as control. Resultant interaction patterns were inspected after a post-production minimization process. Also confirmation of positional and conformational tendency from minimized average structures sampled from the last 1 ns of the simulation (500,000 frames) was regarded. For brevity, only 5NS are shown in Figure 9. In Figures 10 and 11, the HB count along the trajectory are shown to illustrate the strength and stability of the different specific HB interactions of the hits in comparison to reference ligands.

In general the control ligands of the palm region showed a preserved placement (Figure 10 shows HB pattern along the trajectories of palm site complexes), 2JC1 had alternating interactions between ARG386 and TYR448, while for 3D5M a minor backbone rotation has led to hydrogen bonding with SER556, GLN446, and additional TYR448 contact, also two new π-interactions were noticed inside the hydrophobic pocket (π-π with TYR415 and cation-π with ARG386) in a manner baring more resemblance to the original PDB coordinates (rather than the docked one).

A conserved mode was noticed for hit DB05039 as its hydrophobic contact which evolved into more subtly relaxed rotomer was preserved, also the π-π interaction with TYR415, while the an extra HB was formed with the latter. Moreover, DB01940 has undergone significant conformational changes around the site releasing some strain to a more extended conformation, the conformational leap could be noticed in the form of a transient sharp increase in HB count around 2.7 ns, new HB with TYR448, SER556 and a cation-π with ARG158, and less frequently with hydrogen bonding with GLY557 and VAL284.

Calculating the average structure proved that although hit DB00560 possesses a seemingly favourable hydrogen bonding trend, yet the hydrophilic nature of the hit has led to a sufficient binding liability and more favourable ligand-solvent interactions. Notably hit DB04142 has undergone slight rotation to reorient forming an average of 3 hydrogen bonds 2 of which with the TYR448 (the most commonly redundant pharmacophoric feature amongst the analysed PDB's), SER368 and GLN446.

The results of the dynamics simulations for the thumb II site were inherently different due to its nature. The hydrogen bonding pattern seemed very labile owing to the extensive solvent exposure and the shallowness of the binding site, hence the obvious lower density of the resultant HB contacts along the ensemble (Figure 11).

The HB count of hit DB04205 (Figure 11) seems to have dropped suddenly by the end of the first 2.5 ns of the production phase, where a transient partial binding has taken place at the edge of the site hinged through the two phosphate groups till beyond the 4.5 ns, then the ring -by the end of the 5 ns interval- has flipped outwards to form new hydrogen bonds with the surface residues ARG380 and LYS379.

DB01087 has exhibited a very stable trend of hydrogen bonding (Figure 11). Albeit around an average of 1.6 HB, the compound formed two additional π interactions; nominally σ-π with ALA529, and π-π with ARG501 thus sandwiching the substituted electron-rich quinoline scaffold. On the contrary, and as expected hit DB00816 failed to prove any appreciable binding properties and was gradually expelled towards the edge of the binding site.

Thus, from the discussion above, hits DB05039, DB01940, and DB04142 (Figure 6B, C, and 6D show their binding poses and interactions, and Figure 10 shows their Hydrogen bond stability along the MD simulation) would be expected to bare an inhibitory potential against NS5B at the palm region, while for the thumb II site only DB001087 (Figure 8-C) would be prospected to bare an inhibitory potential from amongst the other hits. These virtual hits are currently experimentally tested on the HCV replicon system as well as enzymatic assays.

## Conclusion

The goal of the receptor-based workflow was to identify potential NS5B inhibitors through virtually screening of the Drug Bank database, followed by hit refinement through another more computationally stringent docking stage. Selected hits and three inhibitors from palm and thumb II sites have been subject to 20 ns of explicit-solvent, fully unrestrained molecular dynamics simulations, for which binding patterns were analysed to obtain an insight considering various physical factors -mainly receptor flexibility and solvent effects (Figures show 5NS for brevity). As a result the hits were better characterised according to their binding capabilities and modalities. Eventually, four different hits were recognized to exhibit sufficiently favourable binding properties to be prospected as potential inhibitors of NS5B.

We have generated the common trends of receptor-ligand interactions pharmacophores, calculated from 37 PDB coordinates compiled according to the corresponding sites. Categorizing the palm region into three sub-sites, it seemed that hydrogen bonding with the backbone amide of TYR448 and hydrophobic interaction with the deep pocket were found to be a common binding feature among all chemical subclasses of palm I inhibitors, also polar interaction with ASN316 was common between palm II and palm III inhibitors. While SER476, TYR477 and ARG501 polar contacts constituted the major features for thumb II inhibitors, ARG503 formed the only common polar interaction for thumb I inhibitors. These "essential" interactions that have been defined here constitute a method of selection that could be used in various virtual screening exercises on the NS5B protein. This concept of polypharmacology could be utilised for various new drug discovery endeavours. Reuse of already available safe compounds should thus shorten and lower the expenses of the long drug discovery cycle.

The novel idea of basing a machine-learning activity prediction model on interaction simulation scores was used here for ranking the retrieved hits from first-stage docking with Surflex. It could be further improved as a screening tool, or including essential pharmacophoric interaction data as features in the future.

## Competing interests

The authors declare that they have no competing interests.

## Authors' contributions

ME proposed the study and designed it;ME MG & MF performed the study; ME MG & MF wrote the paper and ME revised the paper, ME and MF edited the paper.

## Supplementary Material

Additional file 1 **Docking Scores and interactions for all NNI binding sites using Surflex-Dock hits across all site sorted in descending order according to Surflex-Dock total score**. Click here for file

Additional file 2 **HCV NS5B ThumbII Binding DB and another dataset training and correlation of different scores and of neural-network model (PIC)**. The model improved the correlation to0.87. Click here for file

Additional file 3 **Neural-network Model implementation on Palm I candidates obtained from Surflex screening on the drug bank**. Click here for file

Additional file 4 **Neural-network model implementation on Thumb II candidate hits obtained from first-stage screening with Surflex docking**. Click here for file
