# Supplementary material for: Multiple virtual screening approaches for finding new Hepatitis c virus RNA-dependent RNA polymerase inhibitors: Structure-based screens and molecular dynamics for the pursue of new poly pharmacological inhibitors
Source: BMC Bioinformatics. 2012 Dec 7;13(Suppl 17):S5. doi: 10.1186/1471-2105-13-S17-S5 (PMC3521232; doi:10.1186/1471-2105-13-S17-S5)
Supplement: Additional file 1 — Docking Scores and interactions for all NNI binding sites using Surflex-Dock hits across all site sorted in descending order according to Surflex-Dock total score. [file 1471-2105-13-S17-S5-S1.doc]

Supplementary Table 1: Docking Scores and interactions for all NNI binding sites using Surflex-Dock hits across all site sorted in descending order according to Surflex-Dock total score.

| Generic Name | Drug Bank Id | Surflex-dock total score | Site |
| --- | --- | --- | --- |
| Cilostazol | DB01166 | 12.16 | Palm |
| 4-Oxoretinol | DB02699 | 10.29 | Palm |
| Propiomazine | DB00777 | 9.98 | thumb2 |
| P-Nitrophenol | DB04471 | 9.65 | Palm |
| CRA_23653 | DB04246 | 9.6 | Palm |
| pradefovir mesylate | DB05478 | 9.55 | Palm |
| Sumatriptan | DB00669 | 9.54 | Palm |
| Doxazosin | DB00590 | 9.4 | Palm |
| CRA_10655 | DB02526 | 9.37 | Palm |
| Anileridine | DB00913 | 9.37 | Palm |
| 2-Phenyl-1-[4-(2-Piperidin-1-Yl-Ethoxy)-Phenyl]-1,2,3,4-Tetrahydro-Isoquinolin-6-ol | DB04471 | 9.36 | thumb2 |
| N-Acetyl Serotonin | DB04275 | 9.35 | Palm |
| 9-(4-Hydroxybutyl)-N2-Phenylguanine | DB02495 | 9.33 | Palm |
| HCV-086 | DB05884 | 9.29 | Palm |
| Indacaterol | DB05039 | 9.23 | Palm |
| Penbutolol | DB01359 | 9.22 | Palm |
| Propidium | DB02166 | 9.19 | Palm |
| Primaquine | DB01087 | 9.13 | Palm |
| PARP inhibitor | DB05940 | 9.11 | Palm |
| Compound 4-D | DB03742 | 9.1 | Palm |
| Almotriptan | DB00918 | 9.07 | Palm |
| Naratriptan | DB00952 | 8.94 | Palm |
| Dapoxetine | DB04884 | 8.87 | Palm |
| Thymidine-3',5'-Diphosphate | DB04205 | 8.83 | thumb2 |
| Methyl-[4-(4-Piperidine-1-Ylmethyl-Phenyl)-Cyclohexyl]-Carbaminic Acid-(4-Chlorophenyl)-Ester | DB03748 | 8.79 | Palm |
| Bifeprunox | DB04888 | 8.76 | Palm |
| Darifenacin | DB00496 | 8.75 | Palm |
| Rbt205 Inhibitor | DB03777 | 8.64 | Palm |
| Indole Naphthyridinone | DB01691 | 8.64 | Palm |
| 2,4-Diamino-6-[N-(3',4',5'-Trimethoxybenzyl)-N-Methylamino]Pyrido[2,3-D]Pyrimidine | DB02919 | 8.63 | Palm |
| Tolterodine | DB01036 | 8.62 | Palm |
| Zanapezil | DB04859 | 8.58 | Palm |
| 3-(4-Amino-2-Tert-Butyl-5-Methyl-Phenylsulfanyl)-6-Cyclopentyl-4-Hydroxy-6-[2-(4-Hydroxy-Phenyl)-Ethyl]-5,6-Dihydro-Pyran-2-One | DB04298 | 8.55 | Palm |
| 9-Butyl-8-(4-Methoxybenzyl)-9h-Purin-6-Amine | DB03899 | 8.55 | Palm |
| Gentian Violet | DB00406 | 8.55 | thumb2 |
| CRA_10656 | DB04470 | 8.53 | Palm |
| Balanol Analog 2 | DB01940 | 8.46 | Palm |
| 3-(3,5-Dibromo-4-Hydroxy-Benzoyl)-2-Ethyl-Benzofuran-6-Sulfonic Acid Dimethylamide | DB04142 | 8.44 | Palm |
| 4-[4-(1-Amino-1-Methylethyl)Phenyl]-5-Chloro-N-[4-(2-Morpholin-4-Ylethyl)Phenyl]Pyrimidin-2-Amine | DB02491 | 8.43 | Palm |
| Valaciclovir | DB00577 | 8.43 | Palm |
| Pheniramine | DB01620 | 8.41 | Palm |
| Lasofoxifene | DB06202 | 8.4 | thumb2 |
| Lasofoxifene | DB06202 | 8.4 | Palm |
| Abacavir | DB01048 | 8.39 | Palm |
| N6-(2,5-Dimethoxy-Benzyl)-N6-Methyl-Pyrido[2,3-D]Pyrimidine-2,4,6-Triamine | DB02583 | 8.36 | Palm |
| Primaquine | DB01087 | 8.36 | thumb2 |
| Darifenacin | DB00496 | 8.36 | thumb2 |
| Farnesol | DB02509 | 8.35 | Palm |
| 2-(Pyrido[1,2-E]Purin-4-Yl)Amino-Ethanol | DB01994 | 8.35 | Palm |
| Donepezil | DB00843 | 8.31 | Palm |
| 1-Benzyl-3-(4-Methoxy-Benzenesulfonyl)-6-Oxo-Hexahydro-Pyrimidine-4-Carboxylic Acid Hydroxyamide | DB04140 | 8.3 | thumb2 |
| Droperidol | DB00450 | 8.3 | thumb2 |
| Aripiprazole | DB01238 | 8.29 | Palm |
| Raloxifene | DB00481 | 8.29 | thumb2 |
| Almotriptan | DB00918 | 8.28 | Palm |
| Orciprenaline | DB00816 | 8.25 | thumb2 |
| Zanapezil | DB04859 | 8.24 | thumb2 |
| Nicergoline | DB00699 | 8.24 | Palm |
| 2'3'-Dideoxyinosine | DB02392 | 8.2 | Palm |
| Cyclopentolate | DB00979 | 8.19 | Palm |
| Bifonazole | DB04794 | 8.12 | Palm |
| Diphenidol | DB01231 | 8.12 | Palm |
| Nialamide | DB04820 | 8.09 | Palm |
| 6-[3-(4-Morpholinyl)Propyl]-2-(3-Nitrophenyl)-5-Thioxo-5,6,-Dihydro-7h-Thienol[2',3':4,5]Pyrrolo[1,2-C]Imidazol-7-One | DB03507 | 8.09 | Palm |
| Nadolol | DB01203 | 8.08 | Palm |
| (4-sulfamoyl-phenyl)-thiocarbamic acid O-(2-thiophen-3-yl-ethyl) ester | DB03333 | 8.06 | Palm |
| ACCLAIM | DB05252 | 8.03 | Palm |
| 4-Sulfonamide-[1-(4-Aminobutane)]Benzamide | DB03697 | 8.03 | Palm |
| (R)-N-(3-Indol-1-Yl-2-Methyl-Propyl)-4-Sulfamoyl-Benzamide | DB02479 | 8.03 | Palm |
| Tolterodine | DB01036 | 8.03 | Palm |
| Zanapezil | DB04859 | 8.02 | Palm |
