# Supplementary material for: Multiple virtual screening approaches for finding new Hepatitis c virus RNA-dependent RNA polymerase inhibitors: Structure-based screens and molecular dynamics for the pursue of new poly pharmacological inhibitors
Source: BMC Bioinformatics. 2012 Dec 7;13(Suppl 17):S5. doi: 10.1186/1471-2105-13-S17-S5 (PMC3521232; doi:10.1186/1471-2105-13-S17-S5)
Supplement: Additional file 2 — HCV NS5B ThumbII Binding DB and another dataset training and correlation of different scores and of neural-network model (PIC). The model improved the correlation to0.87. [file 1471-2105-13-S17-S5-S2.docx]

Su**pplementary Table 2: HCV NS5B ThumbII Binding DB and another dataset training and correlation of different scores and of neural-network model (PIC). The model improved the correlation to0.87.**

| compound |  |  |  |  |  | Glide | MOE | IC50 | PIC50 | Predicted PIC50 |
| --- | --- | --- | --- | --- | --- | --- | --- | --- | --- | --- |
|  | Sybyl Total_score | D_SCORE | PMF_SCORE | G_SCORE | CSCORE | XP GScore |  |  |  |  |
| 409723 | 2.73 | -67.4129 | -35.4728 | -68.6483 | 1 | -7.756541 | -15.5556 | 6200 | -3.79239 | -3.207 |
| 411021 | 3.82 | -72.2378 | 10.4573 | -66.3159 | 0 | -7.710336 | -14.3794 | 4200 | -3.62325 | -3.009 |
| 429706 | 5.12 | -103.288 | 1.6942 | -72.0705 | 3 | -7.551763 | -17.9191 | 9900 | -3.99564 | -2.794 |
| 429721 | 3.67 | -114.356 | -9.8981 | -145.125 | 3 | -7.118768 | -15.072 | 82000 | -4.91381 | -3.471 |
| 441065 | 2.92 | -60.5697 | -33.0361 | -97.7394 | 1 | -6.980253 | -17.1325 | 680 | -2.83251 | -3.411 |
| 441310 | 4.9 | -96.5383 | -8.4442 | -146.634 | 2 | -6.740775 | -18.5696 | 380 | -2.57978 | -2.936 |
| 441282 | 4.4 | -94.2666 | -39.4665 | -192.129 | 3 | -6.372768 | -19.5481 | 230 | -2.36173 | -3.034 |
| 441824 | 5.61 | -118.764 | -14.5709 | -166.635 | 4 | -6.141341 | -17.4839 | 210 | -2.32222 | -3.113 |
| 376757 | 6.32 | -122.756 | -24.211 | -213.823 | 4 | -6.008643 | -13.2719 | 5000 | -3.69897 | -3.985 |
| 377159 | 5.03 | -115.325 | -12.1125 | -190.486 | 4 | -5.78991 | -13.7484 | 14000 | -4.14613 | -4.132 |
| 402238 | 6.87 | -153.966 | -0.3641 | -242.911 | 4 | -5.399422 | -14.1314 | 1450 | -3.16137 | -2.861 |
| 410375 | 4.51 | -118.793 | -17.223 | -148.447 | 4 | -4.901029 | -16.9448 | 15000 | -4.17609 | -3.358 |
| 424082 | 5.94 | -94.5861 | -19.1084 | -28.4795 | 2 | -4.67851 | -14.4123 | 2600 | -3.41497 | -3.427 |
| 424560 | 5.77 | -92.2257 | -83.2544 | 2.1201 | 3 | -4.613487 | -17.4952 | 20000 | -4.30103 | -3.41 |
| 402348 | 6.05 | -133.709 | -33.5069 | -182.999 | 5 | -4.420116 | -12.9283 | 1190 | -3.07555 | -3.628 |
| 6 | 10.1 | -104.312 | -64.4703 | -249.342 | 3 | -7.27 | -19.26 | 3 | -0.47712 | -1.108 |
| 11 | 10.25 | -129.972 | -73.0514 | -281.799 | 5 | -7.208 | -18.396 | 180 | -2.25527 | -0.6 |
| 12 | 10.45 | -158.217 | -70.1316 | -283.878 | 5 | -6.491 | -15.451 | 88 | 1.94448 | -0.681 |
| 13 | 10.47 | -137.428 | -76.974 | -315.958 | 5 | -7.019 | -19.643 | 2 | -0.30103 | -0.717 |
| 14 | 9.53 | -133.955 | -74.303 | -308.993 | 5 | -7.33 | -18.519 | 5 | -0.69897 | -0.703 |
| 15 | 9.65 | -137.433 | -71.6968 | -307.381 | 4 | -6.802 | -17.834 | 140 | -2.14613 | -0.926 |
| 16 | 9.8 | -96.6094 | -78.3754 | -238 | 3 | -8.943 | -15.807 | 10 | -1 | -0.833 |
| 17 | 8.98 | -121.169 | -72.4373 | -268.071 | 4 | -8.249 | -16.971 | 3 | -0.47712 | -0.469 |
| 18 | 11.13 | -126.761 | -75.3531 | -286.9 | 5 | -8.175 | -16.037 | 8 | -0.90309 | -1.095 |
| 19 | 10.08 | -134.376 | -71.1901 | -303.554 | 4 | -8.211 | -16.028 | 6 | -0.77815 | -0.938 |
| 20 | 10.22 | -114.576 | -77.7007 | -286.265 | 5 | -9.027 | -14.714 | 7 | -0.8451 | -0.785 |
| 21 | 9.53 | -84.394 | -73.3192 | -244.678 | 3 | -8.581 | -15.645 | 8 | -0.90309 | -0.707 |
| 22 | 9.71 | -116.997 | -85.4144 | -331.304 | 4 | -9.314 | -15.289 | 10 | -1 | -0.787 |
| 23 | 9.41 | -88.954 | -67.6021 | -291.627 | 3 | -9.325 | -11.586 | 16 | -1.20412 | -1.718 |
| 24 | 10.45 | -127.15 | -78.9155 | -308.8 | 5 | -9.348 | -15.043 | 7 | -0.8451 | -1.033 |
| Correlation | 0.83875 | -0.24801 | -0.75333 | -0.78437 | 0.418247 | -0.644928469 | -0.22203 | -0.56686 | 1 | 0.874873 |
