# Supplementary material for: Multiple virtual screening approaches for finding new Hepatitis c virus RNA-dependent RNA polymerase inhibitors: Structure-based screens and molecular dynamics for the pursue of new poly pharmacological inhibitors
Source: BMC Bioinformatics. 2012 Dec 7;13(Suppl 17):S5. doi: 10.1186/1471-2105-13-S17-S5 (PMC3521232; doi:10.1186/1471-2105-13-S17-S5)
Supplement: Additional file 3 — Neural-network Model implementation on Palm I candidates obtained from Surflex screening on the drug bank. [file 1471-2105-13-S17-S5-S3.docx]

**Supplementary Table 3: Neural-network Model implementation on Palm I candidates obtained from Surflex screening on the drug bank.**

| **Drug Bank Id** | **Drug BankName** | Predicted * -1 | **IC50** |
| --- | --- | --- | --- |
| DB01203 | Nadolol | 1.891 | 78 |
| DB02166 | Propidium | 1.892 | 78 |
| DB00918 | Almotriptan | 1.923 | 84 |
| DB00843 | Donepezil | 1.943 | 88 |
| DB04859 | Zanapezil | 1.97 | 93 |
| DB02919 | 2,4-Diamino-6-[N-(3',4',5'-Trimethoxybenzyl)-N-Methylamino]Pyrido[2,3-D]Pyrimidine | 1.989 | 97 |
| DB01036 | Tolterodine | 2.19 | 155 |
| DB05039 | Indacaterol | 3.962 | 9162 |
| DB04142 | 3-(3,5-Dibromo-4-Hydroxy-Benzoyl)-2-Ethyl-Benzofuran-6-Sulfonic Acid Dimethylamide | 4.944 | 87902 |
| DB01940 | Balanol Analog 2 | 5.626 | 422669 |
