# Supplementary material for: Multiple virtual screening approaches for finding new Hepatitis c virus RNA-dependent RNA polymerase inhibitors: Structure-based screens and molecular dynamics for the pursue of new poly pharmacological inhibitors
Source: BMC Bioinformatics. 2012 Dec 7;13(Suppl 17):S5. doi: 10.1186/1471-2105-13-S17-S5 (PMC3521232; doi:10.1186/1471-2105-13-S17-S5)
Supplement: Additional file 4 — Neural-network model implementation on Thumb II candidate hits obtained from first-stage screening with Surflex docking. [file 1471-2105-13-S17-S5-S4.docx]

**Supplementary Table 4: Neural-network model implementation on Thumb II candidate hits obtained from first-stage screening with Surflex docking.**

| Name | ID | Sybyl Total_score | Fitness | glide_constrained | MOE | PIC50 | IC50 |
| --- | --- | --- | --- | --- | --- | --- | --- |
| Thymidine-3',5'-Diphosphate | DB04205 | 8.83 | 68.5 | -6.52423 | -18.38 | -1.011 | 10.25652 |
| Orciprenaline | DB00816 | 8.25 |  | -9.08986 | -15.92 | -1.212 | 16.29296 |
| 1-Benzyl-3-(4-Methoxy-Benzenesulfonyl)-6-Oxo-Hexahydro-Pyrimidine-4-Carboxylic Acid Hydroxyamide | DB04140 | 8.3 | 74.25 | -7.13225 | -15.67 | -1.491 | 30.97419 |
| 2-Phenyl-1-[4-(2-Piperidin-1-Yl-Ethoxy)-Phenyl]-1,2,3,4-Tetrahydro-Isoquinolin-6-Ol | DB04471 | 9.36 | 63.14 | -6.55447 | -12.2 | -1.914 | 82.03515 |
| Lasofoxifene | DB06202 | 8.4 | 62.96 | -7.23998 | -12.33 | -2.012 | 102.8016 |
| Raloxifene | DB00481 | 8.29 | 71.79 | -2.933 | -15.14 | -2.127 | 133.9677 |
| Primaquine | DB01087 | 8.36 | 51.7 | -6.10334 | -10.79 | -2.428 | 267.9168 |
| Darifenacin | DB00496 | 8.36 | 64.87 | -4.90635 | -11.12 | -2.522 | 332.6596 |
| Propiomazine | DB00777 | 9.98 | 62.49 | -0.98499 | -11.48 | -2.62 | 416.8694 |
| Zanapezil | DB04859 | 8.24 | 64.82 | -5.87869 | -9.39 | -2.708 | 510.505 |
| Droperidol | DB00450 | 8.3 | 64.88 |  | -8.91 | -3.309 | 2037.042 |
